# Comparative Analysis of Two Zika Virus Isolates in a Rhesus Macaque Pregnancy Model

**DOI:** 10.3390/v17060762

**Published:** 2025-05-27

**Authors:** Hannah K. Jaeger, Jessica L. Smith, Christopher J. Parkins, Nicole N. Haese, Craig N. Kreklywich, Michael Denton, Caralyn S. Labriola, Michael K. Axthelm, Aaron Barber-Axthelm, Kim Chun, Tonya Swanson, Rahul J. D’Mello, Terry K. Morgan, Duncan R. Smith, Jamie O. Lo, Alec J. Hirsch, Victoria H. J. Roberts, Daniel N. Streblow

**Affiliations:** 1 The Vaccine & Gene Therapy Institute, Oregon Health and Science University, Beaverton, OR 97006, USA; jaegerh@ohsu.edu (H.K.J.); hirschal@ohsu.edu (A.J.H.); 2 Division of Pathobiology & Immunology, Oregon National Primate Research Center, Oregon Health and Science University, Beaverton, OR 97006, USA; 3 Division of Reproductive & Developmental Sciences, Oregon National Primate Research Center, Oregon Health and Science University, Beaverton, OR 97006, USArobertsv@ohsu.edu (V.H.J.R.); 4 Department of Obstetrics & Gynecology, Oregon Health & Science University, Portland, OR 97239, USA; 5 Division of Pathology, Oregon Health & Science University, Portland, OR 97239, USA; 6 Institute of Molecular Biosciences, Mahidol University, Salaya Campus, 25/25 Phuttamonthol Sai 4, Nakhon Pathom 73170, Thailand; duncan_r_smith@hotmail.com

**Keywords:** Zika virus, congenital Zika syndrome, non-human primate, placenta, immunology, Thailand

## Abstract

Zika virus (ZIKV) infection during pregnancy can cause a broad range of neurological birth defects, collectively named Congenital Zika Syndrome (CZS). We have previously shown that infection with the Puerto Rican isolate PRVABC59 (ZIKV-PR) results in abnormal oxygen transport in the placenta due to villous damage and uterine vasculitis in a nonhuman primate model. To investigate whether this type of damage occurs with endemically circulating strains in Thailand, we investigated a CZS case isolate, MU1-2017 (ZIKV-TH), in pregnant rhesus macaques. Pregnant animals (*n* = 3 per group) were infected subcutaneously with either ZIKV-PR or ZIKV-TH at ~50 days gestation (GD) and monitored for 40 days post-infection (GD90). Similar courses of viremia and immune activation were observed for both viruses when compared to uninfected controls. In addition, both viruses induced changes to the placental architecture, including spiral artery remodeling and the development of infarctions. Similar levels of viral RNA were detected at necropsy in maternal and fetal tissues. Overall, our results show that the ZIKV-TH strain MU1-2017 behaves similarly to the ZIKV-PR strain, and, importantly, provide evidence of in-utero infection with an additional contemporary strain of ZIKV.

## 1. Introduction

Zika virus (ZIKV) is an *Orthoflavivirus* that is primarily transmitted through the bite from infected *Aedes aegypti* mosquitoes [1]. First isolated in Africa in 1947, ZIKV was subsequently isolated in Southeast Asia. Phylogenetic analysis classifies ZIKV as two distinct lineages: African and Asian that have been expanded to include the Asian American clade [2,3,4]. The spread of Asian lineage ZIKV stains through the South Pacific and into South America in 2013–2016 resulted in an explosive outbreak. Although infection is generally subclinical in healthy individuals, one in five people experience febrile illness or a combination of headaches, myalgia, rash, or conjunctivitis [5]. Most alarming, however, is the observation that in pregnant women ZIKV can cause congenital birth defects or even fetal demise, with a phenotype collectively termed as Congenital Zika Syndrome (CZS) [6,7]. Data from the Brazil outbreak revealed that 46% of pregnancies affected by ZIKV resulted in severe neurological birth defects [6]. Although the incidence of ZIKV cases has declined since then, sporadic outbreaks and ongoing cases persist in the Americas and other regions of the world, underscoring the risk of future re-emergence [8,9].

ZIKV is endemic to several countries in Southeast Asia, including Thailand. Despite genetic similarities between the Brazilian and Thai ZIKV strains [2,10], an epidemic with high rates of congenital infection has not previously been reported in Thailand [11]. Serologic evidence indicates ZIKV circulation as early as 2002 [12,13,14], with recent studies showing low-level ongoing transmission and a significant level of immunity in the Thai population [11]. New cases have been rising in Thailand with 750 people infected and at least 13 out of 33 pregnancies resulting in babies born with birth defects in 2023 [15]. Recent reports indicate that there are two distinct clades of ZIKV circulating within Thailand that include the Asian-American and the Southeast Asian strains [16]. Infections with some strains are associated with rapid viremia and fever induction (SV0127/14), while others, such as the CZS isolate from 2017 (MU1-2017), exhibit stronger fetal tropism. Differences in growth kinetics between these strains may contribute to variations in pathogenicity [17].

Several animal studies have explored this topic and pointed to strains either gaining fetal tropism through mutations, or specific strains being more pathogenic. Notably, the prM S139N (prM S13N) mutation—linked to increased neurotropism and first identified during the 2013 French Polynesian outbreak—is present in many South American strains, including ZIKV PRVABC59 (ZIKV-PR) but is absent in the MU1-2017 (ZIKV-TH) isolate [18]. There are several other mutations, including NS2B I39V, which are linked to increasing vertical transmission in mice that both strains retain, indicating that several factors could contribute to increased fetal pathogenesis [19]. The MU1-2017 strain was isolated in Bangkok in 2017 from autopsy specimens of a fetus medically terminated for suspected CZS. PCR analysis showed the presence of ZIKV in the placenta, cerebrum, umbilical cord, brain stem, skin muscle, and bone marrow. The virus was recovered from both brain stem and cerebrum. Furthermore, this strain demonstrates distinct replication kinetics compared to ZIKV strains linked to febrile illness in Thailand, suggesting that maternal–fetal transmission may be influenced more by viral replication kinetics than specific mutations [17]. Whether there are biological differences that affect outcomes during pregnancy between endemic Thai strains and South American strains remains unclear. Therefore, further investigation is needed to assess differences in fetal tropism, congenital outcomes, and placental pathology among ZIKV strains.

Non-human primates (NHPs) offer a robust model for studying ZIKV-induced obstetric complications due to their physiological and immunological similarities to humans. Studies using NHP models provide a unique opportunity to evaluate the timing of viral pathogenesis, placental pathology, and fetal infection [20,21,22,23,24,25,26,27,28,29]. NHPs infected with the Asian-lineage ZIKV strains result in ~26% of fetal loss when pregnant macaques were inoculated during the first trimester, which recapitulates what has been reported in humans [30]. There is a range of adverse outcomes within the spectrum of anomalies observed with CZS, many of which can occur in the absence of severe neonatal central nervous system (CNS) deficits [25,31,32,33]. These outcomes are seen in both humans and NHPs and include spontaneous abortions, stillbirth, fetal infection, intrauterine growth restriction (IUGR), oligohydramnios, preterm premature rupture of membranes, and preterm delivery [34,35]. Although research since the 2015 epidemic has largely focused on vector control, vaccine development, and neurological complications, the broad spectrum of ZIKV-associated pregnancy complications highlights the importance of understanding the role of the placenta in mediating these outcomes.

Our group has utilized ultrasound technology to study the relationship between utero–placental blood flow dynamics with placental pathology and ZIKV-induced damage using a rhesus macaque model. Previous work has shown that first-trimester infection leads to more severe outcomes, consistent with findings from other NHP studies. Until now, our studies have used the 2015 Puerto Rican isolate (ZIKV-PR, PRVABC59), which is genetically similar to the Brazilian isolates from the same outbreak period. Non-invasive ultrasonography provides real-time, quantitative measurements of fetal biometry, placental ultrastructure, and maternal spiral artery and fetal umbilical artery flux rates that can be assessed throughout pregnancy [36]. Using serial prenatal ultrasound (US) assessments, increased placental echogenicity in all ZIKV-infected dams was observed, suggestive of placental ischemic injury leading to placental insufficiency [37,38]. Contrast-enhanced US assessment of the placental intervillous space was notable for a significant increase in flux rate suggestive of the increased velocity of maternal blood delivered by the maternal spiral arteries that may result in shear stress-induced placental injury to the villi. These ultrasound findings are consistent with reported placental damage and an increased incidence of miscarriage in human pregnancies infected with ZIKV [30,39]. Histologic examination from multiple studies has confirmed these US findings, demonstrating increased fibrin deposition around spiral arteries, increased villous damage, gross infarctions, calcifications, and sporadic detection of diffuse ZIKA RNA [40,41,42,43,44,45,46].

Given that ZIKV-TH was isolated from fetal brain tissue in a case of congenital Zika syndrome (CZS), whereas ZIKV-PR (PRVABC59) was derived from maternal serum, we hypothesized that ZIKV-TH would result in higher rates of congenital abnormalities and placental dysfunction. To investigate strain-specific effects on pregnancy outcomes, rhesus macaques were infected during the first trimester and monitored for 40 days. Comprehensive assessments included longitudinal viremia and immune profiling through routine blood draws, alongside serial non-invasive US evaluations to track fetal growth, uteroplacental blood flow, and amniotic fluid index. By directly comparing the pathogenicity of ZIKV-PR and ZIKV-TH, this study aimed to provide critical insights into viral strain-specific impacts on pregnancy and placental health.

## 2. Materials and Methods

### 2.1. Animal Ethics Statement

All experimental protocols were approved by the Institutional Animal Care and Use Committee (IACUC) of the Oregon National Primate Research Center (ONPRC), which is accredited by the Assessment and Accreditation of Laboratory Animal Care (AAALAC) International. All procedures in this study complied with the ethical standards outlined in the Animal Welfare Act, as enforced by the United States Department of Agriculture, and followed national guidelines for the care and use of laboratory animals. The study also adheres to the ARRIVE guidelines for animal research [47].

### 2.2. Zika Virus (ZIKV) Preparation and Cell Culture

Zika virus PRVABC59 (ZIKV-PR) was isolated by the Centers for Disease Control (CDC) from an individual in Puerto Rico in December 2015 [4]. ZIKV-TH was recovered from autopsy specimens of a fetus medically terminated for suspected CZS in Thailand (strain ZIKV MU1-2017) [17], which is genetically identical to the independently isolated SI-BKK02 strain (GenBank accession no. MF996804) [48]. The strains used in these studies were re-isolated by transfection of viral RNA into Vero cells. The resulting viral stocks were subsequently passaged twice in C6/36 mosquito cells (ATCC CRL-1660) to maintain viral genomic integrity and minimize the risk of adaptive mutations that can arise from repeated passaging in mammalian cells [38,49].

**Figure 1:**
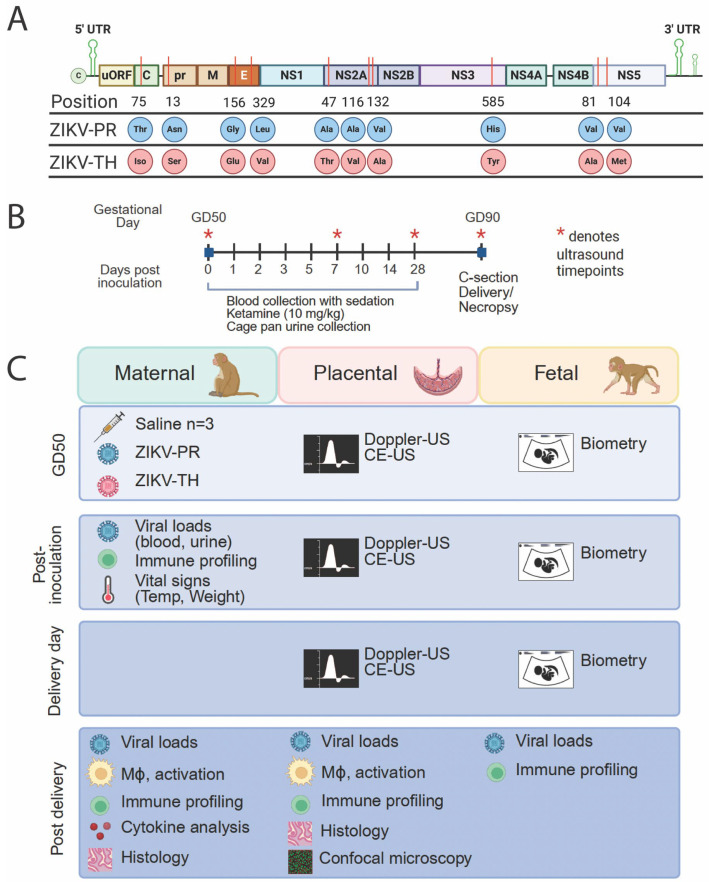
Study design and viral phylogeny of viruses used in non-human primate studies. (**A**) Viral genome alignment between PRVABC59 and MU1-2017 (MF996804) was performed using Benchling (https://www.benchling.com/, accessed on 30 November 2024) to assess sequence identity and variations. The final alignment was based upon sequencing performed post-inoculation, using virus isolated from rhesus macaque tissues. Positions are listed based on the beginning of each gene. (**B**) Nine rhesus macaque pregnancies were grouped into either ZIKV-inoculated or controls. On gestational day (GD) 50 (±2 days), animals were exposed subcutaneously (SQ) to either saline, ZIKV-PR, or ZIKV-TH. (**C**) Viral loads, immune cell profiling, antibody kinetics, and vital signs were monitored longitudinally for each group. Maternal–fetal interface (MFI) and fetal/embryonic tissues were collected on the date of necropsy at GD90 via C-section. Graphics were created in BioRender. Jaeger, H. K. (2025) https://BioRender.com/840g793 (accessed on 28 April 2025).

The working stocks were concentrated by ultracentrifugation through a 20% sorbitol cushion and titered on confluent monolayers of Vero cells (ATCC CRL-1586). Both viruses were sequenced to confirm phylogeny [49] and found to conform to the previously described sequence for ZIKV-PR (GenBank accession #KU501215.1) and ZIKV-TH (GenBank accession no. MF996804). All amino acid changes are summarized in Figure 1A. All cells were cultured in Dulbecco’s modified Eagle Medium (DMEM) containing 2 mM l-glutamine (Invitrogen, Carlsbad, CA, USA), 100 U/mL penicillin G-sodium, and 100 µg/mL streptomycin sulfate (1X PSG; Invitrogen), and 5–10% fetal calf serum (FCS).

### 2.3. Pregnant Dams

Female rhesus macaques (*n* = 9) used in this study were all healthy, average-weight animals that were fed a standard chow diet (Purina Mills), had access to water ad libitum and assigned to the ONPRC time-mated breeding (TMB) program. The cohort size was determined based on our initial Zika study and on prior NHP studies by our group using similar assessments [37,38]. Similar to previous studies, daily blood sampling was used to coordinate the breeding attempts at 7 days prior to the estradiol surge. Dams were housed in female pairs with continuous full contact, except for the time (typically 3–5 days) when females were transferred and co-housed with the male breeder for time-mated breeding. All pregnancies were identified early by daily hormone monitoring and confirmed by prenatal ultrasound. The timed mating scheme allowed for accurate age determination within 24–48 h of conception. At gestational day (GD) 50, pregnant rhesus macaques were inoculated subcutaneously with a total of 10^5^ focus forming units (ffu) of ZIKV-PR or ZIKV-TH that were diluted in 1 mL of normal saline. The inoculum was distributed as ten total 100 µL injections into both hands, wrists, and lower arms. Injections were administered at the radial and medial aspects of the wrist, the dorsal (back) hand in both radial and medial positions, and one injection near the mid-dorsolateral antebrachium on each arm. This anatomical distribution was selected to promote uniform subcutaneous absorption and systemic dissemination of the virus, while ensuring consistency across all experimental and control animals. Control dams were inoculated with 1 mL of saline in the same manner as the ZIKV-exposed animals.

The animals were monitored daily for clinical signs of disease or discomfort. As depicted in Figure 1B, ultrasound studies were performed to monitor fetal health and development on 0, 7, 28, and 40 dpi, which are the same days that body temperature and weight were measured. Blood and urine were collected on days 0, 1, 2, 3, 5, 7, 10, 14, 21, 28, and 40. These procedures were conducted in accordance with established protocols at our institution to minimize stress and adverse outcomes [36,38]. All animals were monitored closely for changes in behavior, appetite, and weight. No signs of maternal distress or fetal loss were observed that could be attributed to the anesthesia or fasting regimen.

Whole blood was layered over a lymphocyte separation medium (Corning, Corning, NY, USA) and centrifuged for 40 min at 2000 rpm for plasma and peripheral blood mononuclear cell (PBMC) isolation. PBMCs intended for cellular phenotyping analyses were washed in RPMI medium (Fisher) supplemented with 5% FBS and 1X PSG and frozen in liquid nitrogen at ~2 million cells/mL in a freezing medium containing 10% DMSO/40% FBS/50% RPMI. ZIKV-infected pregnant dams were humanely euthanized after cesarean-section delivery at GD90 (40 dpi), and complete necropsies were performed. Representative tissue sections (~1 cm^3^) collected from both dams and fetal muscle, joints, lymphoid tissues, major organs, nervous system, and reproductive organs were collected into 1 mL TRIzol reagent (Invitrogen, Carlsbad, CA, USA) for RNA isolation or fixed in 10% formalin for histopathology. An additional section from each tissue was preserved in RNAlater for future studies. Control dams were delivered via a C-section at ~GD90 and then released from the study.

### 2.4. Viral RNA Detection

RNA from tissue samples, blood, urine, and cerebrospinal fluid (CSF) was isolated using the Zymo Research (Irvine, CA, USA) Direct-zol RNA Miniprep Kit (Cat. R2050) according to the manufacturer’s protocol. Tissue sections were collected into tubes containing 1 mL of TRIzol and approximately 250 µL of silica beads (VWR 48300–437). The samples were homogenized using a Precellys 24 homogenizer (Bertin Technologies, Paris, France) pulsing for three cycles of 45 s on and 30 s off. ZIKV RNA levels were measured by a one-step quantitative real-time reverse transcription polymerase chain reaction assay (qRT-PCR) using TaqMan One-Step RT-PCR Master Mix (Applied Biosystems, Waltham, MA, USA), as previously described [49]. Primer/probe sequences for the detection of ZIKV-PR include the following: forward: 5′-TGCTCCCACCACTTCAACAA; reverse: 5′TGA GGCAGGGAACCACAATGG; and TaqMan probe: 5′ Fam-TCCATCTCAAGGACGG -MGB. Primer/probe sequences for the detection of ZIKV-TH include the following: forward: 5′-TGCTCCCACCATTTCAACAA; reverse: 5′GGCAGGGAACCACAATGG; and TaqMan probe: 5′ Fam-TCCATCTCAAGGACGG-MGB. For RNA standards, RNA was isolated from purified, titered stock of ZIKV-PR or ZIKV-TH.

### 2.5. ZIKV Enzyme-Linked Immunosorbent Assay (ELISA)

ELISAs were performed using high-binding 96-well plates (Corning, Corning, NY, USA) coated with 1 × 10^6^ ffu per well of purified ZIKV-PR, due to the conserved sequence similarity of the main antigenic target E protein, diluted in PBS overnight [38,49]. Plates were blocked with 5% milk/PBS + 0.05% Tween and then incubated with five-fold dilutions of heat-inactivated plasma starting at a dilution of 1:50. HRP-conjugated secondary antibody (anti-monkey IgG or anti-monkey IgM; Rockland, Limerick, PA, USA) was used at 1:1000 and detected using the OPD substrate (0.05 M citrate, 0.4 mg/mL o-phenylenediamine, 0.01% hydrogen peroxide, pH 5) from Life Technologies, followed by 1M HCl after 5–7 min to stop the assay. The plates were read within 10 min using a Synergy HTX Microplate Reader (BioTek, Winooski, VT, USA) at 490 nm. A Log/Log transformation method was used to determine the endpoint titer, and the results were analyzed and visualized using GraphPad Prism v10.2 software.

### 2.6. Focus Reduction Neutralization (FRNT) Assays

FRNT assays were performed as previously described [38]. Five-fold serial dilutions of heat-inactivated plasma samples were made in 2% FBS/DMEM starting at 1:20 and mixed with 200 ffu/well of ZIKV-PR for 1–2 h at 37 °C. The mixture was then added to Vero cells seeded into 96-well plates and incubated for 1 h with rocking before overlaying with CMC/2% FBS/DMEM. Plates were fixed at 30 h post-infection with 4% paraformaldehyde and blocked with 0.05% Tween 20 in PBS for 1 h. To detect focus-forming units, the plates were stained with the anti-flavivirus envelope monoclonal antibody 4G2 (ATCC:HB-112) [50] and followed by secondary antibody anti-mouse IgG-HRP (Santa Cruz Biotech, Dallas, TX, USA sc2005). Foci were visualized by development with VIP substrate (Vector labs, Newark, CA, USA) and counted using the ELISpot reader (AID, Strassburg, Germany). The 50% focus neutralization titers (FRNT50) were calculated by non-linear regression using GraphPad Prism v10.2 software after determining the percentage of foci at each dilution relative to control wells containing no plasma.

### 2.7. Phenotypic Analysis of Peripheral Blood Mononuclear Cells

Flow cytometry was performed to phenotype maternal peripheral blood mononuclear cells (PBMCs), as previously described [37,38,51]. Approximately 1 × 10^6^ PBMC were thawed per phenotypic panel and resuspended in RPMI medium supplemented with 10% FBS and 1X PSG. Cells were pelleted at 2000 rpm and washed with 1X PBS. For innate immune cell analysis, cells were stained using fluorophore-conjugated antibodies directed against the cell surface markers CD45, CD3, CD8a, CD14, CD16, CD11c, HLA-DR, CD56, CD123, and CD169 (activation marker). Monocytes and macrophages were defined as CD3–/CD20–/CD8–/HLA-DR+ with classical monocytes being CD16–/CD14+, intermediate monocytes being CD16+/CD14+, and non-classical monocytes being CD16+/CD14–. Dendritic cells (DCs) were defined as CD3–/CD20–/CD8–/HLA-DR+/CD16–/CD14- with myeloid DCs being CD11c+/CD123– and plasmacytoid DCs being CD11c-/CD123+. T-cells were stained using fluorophore-conjugated antibodies directed against the cellular differentiation markers CD45, CD3, CD4, CD8a, CD25, CD28, CD95, CD127, and intracellular Ki67 and Granzyme B. Naïve CD4+ or CD8+ T cells were defined as CD28+/CD95–, central memory was defined as CD28+/CD95+, and effector memory was defined as CD28–/CD95+. Ki67 and Granzyme B+ populations were gated based on day 0 for each animal. For both panels, CD45+ cells were gated to exclude red blood cell contamination followed by singlet gating before phenotype analysis. Sample analysis was performed using an A528A: BD symphony (BD Pharminogen, San Diego, CA, USA), analyzed with FlowJo v10 software. For innate immune cells, longitudinal changes in total or activated (CD169+) cells in the peripheral blood were analyzed using a mixed model approach with a Gaussian greenhouse correction, followed by Tukey’s multiple comparisons comparing control animals to ZIKV-infected animals for each day post inoculation; **** *p* < 0.0001, *** *p* = 0.0001, ** *p* < 0.001, * *p* < 0.05, with ns *p* > 0.05. A similar analysis was performed for the T-cell panel, looking at Ki67+ or Granzyme B+ in each cellular population subset.

### 2.8. Ultrasound Imaging Studies

Sonographic assessments were performed between gestational days 48 and 90. At the time of imaging studies, animals were sedated via IM injection with 10 mg/kg of ketamine. Animals were then intubated and maintained under general anesthesia with 1% to 2% inhaled isoflurane gas during the imaging study in the ONPRC surgical unit. All imaging procedures were conducted under isoflurane anesthesia following a short fasting period (approximately 6 h), in accordance with standard protocols approved by our Institutional Animal Care and Use Committee (IACUC). Isoflurane was selected due to its well-documented safety profile, characterized by rapid induction and recovery, minimal cardiovascular and respiratory depression, and low metabolic activity in both maternal and fetal tissues. While some studies in pregnant nonhuman primates and other animal models have reported evidence of anesthesia-associated neuroapoptosis, these effects are largely dose- and duration-dependent. When used within standard clinical parameters, as in our study, the risk of lasting fetal harm appears minimal [52,53,54,55]. To further mitigate potential confounding effects, ultrasound imaging was performed only when necessary, and fetal heart rate assessments were conducted while dams were awake whenever possible. Animals were placed in the dorsal recumbent position and physiological vital signs were monitored throughout the procedure by a trained veterinary technician. All ultrasound measurements were collected by a board-certified Maternal–Fetal Medicine physician (J.O.L.) using B-Mode or image-directed pulsed and color Doppler on a GE machine (GE Voluson 730) with a 5- to 9 MHz sector probe. Doppler ultrasound measurements were collected with the lowest high-pass filter level (100 Hz) and an angle of 15° or less between the vessel and the Doppler beam.

### 2.9. Fetal Biometry

Standard fetal biometry measurements were obtained consisting of biparietal diameter (BPD), head circumference (HC), abdominal circumference (AC), and femur length (FL), as described previously [37,38,56]. Head measurements were taken in the trans-thalamic plane. The BPD was measured ‘outer to inner’ using calipers and the HC was measured using the ellipse facility, with the line of the ellipse on the outer border of the skull for the HC. Abdominal circumference measurements were taken with the umbilical vein in the anterior third of a transverse section of the fetal abdomen, at the level of the portal sinus, and the AC was measured using the ellipse facility, placing the line of the ellipse on the outer border of the abdomen. The femur closest to the ultrasound probe was measured for FL with its long axis as horizontal as possible, and calipers were placed on the outer borders of the diaphysis of the femoral bone excluding the trochanter. For fetal biometry graphs, historical control data for 10%, 50%, and 90% was originally calculated using the linear regression and graphed using a published data table from ONPRC [36].

### 2.10. Amniotic Fluid Index

Amniotic fluid volume was assessed as previously described [57]. The uterus was divided into four quadrants and the deepest vertical pocket of fluid, free of umbilical cord or fetal parts, in each of the four quadrants was measured. The amniotic fluid index (AFI) is the summation of the four quadrants.

### 2.11. Uterine Artery Volume Blood Flow

Blood flow velocity waveforms were obtained from the proximal portion of the uterine artery [36,58]. An average of three Doppler waveform measurements for the uterine artery were acquired and the following measurements were obtained: pulsatility index (PI), velocity time integral (VTI), and maternal heart rate (MHR) [36]. The diameter of the uterine artery was measured using power angiography [58]. The cross-sectional area (CSA) of the vessel was calculated as $CSA=\pi {\left(\frac{diameter}{2}\right)}^{2}$ Uterine artery volume blood flow (cQUta) was calculated using the following formula: cQUta = VTI × CSA × MHR. Umbilical artery Doppler velocimetry waveforms were obtained from a free loop of cord with measurements taken in the absence of fetal breathing or body movement [58,59].

### 2.12. Contrast-Enhanced Ultrasound Analysis

At gestational days 50, 57, 78, and 90, following an overnight fast, animals were sedated by intramuscular injection with 10 mg/kg ketamine. Animals were intubated and maintained under anesthesia with 1–2% inhaled isoflurane gas under the supervision of a trained veterinary technician. Contrast-enhanced ultrasound was performed using a multiphase amplitude-modulation and phase-inversion algorithm on a Sequoia system (Siemens Medical Systems, Mountain View, CA, USA) [38,58,60]. A lipid-shelled octofluoropropane microbubble contrast reagent (Definity^®^, Lantheus Medical Imaging, Billerica, MA, USA) was prepared in 0.9% saline at a final concentration of 5% for intravenous infusion via a cephalic catheter. Individually identified maternal spiral artery sources and the microbubbles within the path of the beam were destroyed by a brief (2 s) increase in mechanical index. Microbubble re-entry in the spiral artery and the IVS was recorded at 1 frame/75 ms until the area of interest reached signal saturation (VI_max_). Three replicates of all recordings were obtained during each study and the digital imaging data were analyzed using a custom-designed CE-US analysis program [60]. In brief, regions of interest were traced to obtain the area of each placental cotyledon. The data were fit to the function y = A(1 − e − βt), where y is the VI at the pulsing interval t; A is the VI plateau; and β is the flux rate constant. A one-way ANOVA with Tukey’s post hoc multiple comparison test was used to compare microvascular flux rate data.

### 2.13. Histopathology

Tissues collected at necropsy for histological analysis were fixed in 10% neutral buffer formalin for 24 h and then transferred to 70% ethanol and kept at 4 °C for 24–72 h. The samples were then paraffin-embedded, and slides were cut from the tissue blocks representing the placenta (2 individual 0.5 cm^3^ samples/cotyledon). The slides were stained with hematoxylin and eosin (H&E). Sections from ZIKV-PR (*n* = 3), ZIKV-TH (*n* = 3) and gestational age-matched negative controls (*n* = 3; GD90) were examined by a board-certified gynecologic pathologist (T.K.M.) blinded to exposure groups. The histopathological assessment included scoring for the presence or absence of placental infarctions, accelerated villous maturation, villous stromal calcifications, chronic villitis, acute chorioamnionitis, and chronic deciduitis. Complete cross-sections of decidual spiral arteries were evaluated for luminal dilation and the presence or absence of vasculitis. Tissue slides were examined using Leica DFV495 light microscopes (Wetzlar, Germany) and digitized with the Olympus VS200 scanner (Center Valley, PA, USA) at 20× magnification. Digital images and figures were generated using Halo software version 11.1.

## 3. Results

### 3.1. Study Design

Zika virus infection in pregnant rhesus macaques recapitulates many of the hallmarks of CZS in humans. Table S1 summarizes different ZIKV strains used in NHP pregnancy studies to assess viral pathogenesis and is further discussed in a recent review [22]. During the 2017 outbreak in Thailand, ZIKV was identified in five patients, including three pregnant women. The ZIKV-TH (MU1-2017) strain was isolated from the cerebrum of a terminated fetus at 17 weeks gestational age during this outbreak in Bangkok, Thailand [17]. Our study evaluated the infection and disease outcomes for the ZIKV-TH isolate in pregnant NHPs and compared host and viral outcomes to a ZIKV-Puerto Rican isolate PRABC59 (ZIKV-PR). The Puerto Rican and Thai isolates are both members of the Asian lineage, with 99.7% similarity and a total of 10 amino acid changes in Capsid protein, Pre-Membrane, Envelope, NS2A, N3, and NS5 2-OMTase (Figure 1A). For this study, nine rhesus macaques were divided into three cohorts (Figure 1B,C). Previous studies suggest that infection early in pregnancy has the highest impact on placental dysfunction, with effects developing by 40 dpi [37,38]. With this in mind, we chose gestational day (GD) 50 (range GD 48–52) for infection, equivalent to approximately 90 days in human pregnancy [61] during the end of the first trimester (Figure 1B). Cohort I was inoculated with saline and served as a negative infection control to evaluate normal placental growth and fetal development. Cohorts II and III were inoculated subcutaneously in both hands, wrists, and arms at five sites per arm (100 µL per injection) to mimic a mosquito bite with a total infectious dose of 1 × 10^5^ FFU of either PRVABC59 (ZIKV-PR) or MU1-2017 (ZIKV-TH), with a study endpoint of 40 dpi (GD 90) to assess placental damage and potential congenital infection (Figure 1C).

### 3.2. Comparison of ZIKV Strain Differences in Maternal Viral Loads and Immunity, Fetal Infection and Disease

**Figure 2:**
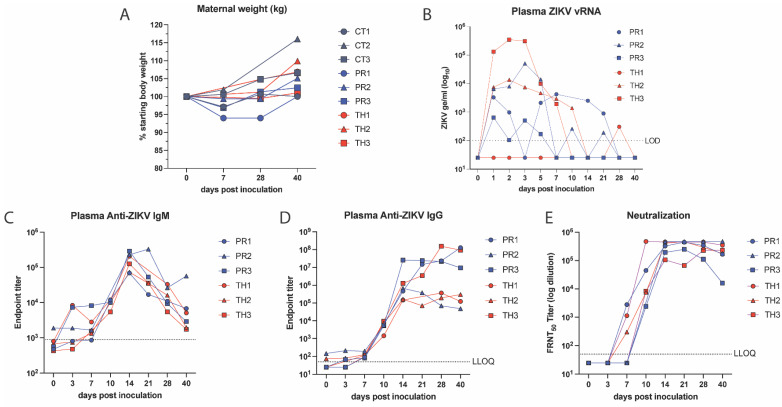
Maternal viremia and characterization of ZIKV-specific antibodies. (**A**) Maternal body weight was measured in kg, graphed as the percentage of starting weight for each animal. (**B**), Maternal plasma viremia was detected via qRT-PCR and is representative of three technical replicates. The LOD was 100 copies of ZIKV RNA per mL of plasma with undetectable samples graphed as 50 copies vRNA/mL plasma. (**C**,**D**) The development of ZIKV-binding IgM and IgG isotype antibody titers was quantified in macaque plasma at indicated timepoints using ELISA. Detection levels at day 0 were used to perform background subtraction (dashed line) to normalize the responses for IgM and the lower limit of detection (LLOQ) for both assays was 1:50. (**E**) The longitudinal development of ZIKV-neutralizing antibodies was quantified in ZIKV neutralization assays using the ZIKV-PR strain as the antigen and heat-inactivated macaque plasma at indicated time points post-inoculation. The 50% focus reduction neutralization titers (FRNT_50_) were determined using non-linear regression and graphed using GraphPad Prism v10.2. The lower limit of quantification was a 1:50 plasma dilution and undetectable values were graphed as half of the LLOQ.

Maternal body weight was measured as a sign of disease and showed a steady increase in ZIKV-TH animals, which mirrored controls, as would be expected during pregnancy (Figure 2A). Compared to the ZIKV-PR, an average decrease in body weight was observed at 7 dpi and weight gain lagged behind until endpoint at 40 dpi. This was particularly pronounced in one animal (PR1) with a 6% reduction in body weight at 7 dpi and 28 dpi before returning to baseline at 40 dpi. The duration of maternal viremia is considered an important factor contributing to transplacental dissemination and could contribute to clinical symptoms such as weight loss [62]. Maternal viremia kinetics were measured by qRT-PCR for both ZIKV-PR- and ZIKV-TH-infected animals. Two of three animals in each group had detectable ZIKV vRNA in plasma by 1-day post-infection (dpi) and reached peak plasma viral load between 1 and 3 dpi (mean time to peak was 2 dpi) (Figure 2B and Figure S1). ZIKV plasma viremia was resolved by 14 dpi in all animals, with sporadic positive detections in three animals at low genome copy numbers at 21 and 28 dpi. To examine the magnitude and duration of ZIKV detection in maternal plasma, the area under the curve (AUC) for each dam’s ZIKV plasma viral load was calculated. The AUC values were compared between strains using a paired two-tailed t-test, which showed that these values did not significantly differ between strains (*p* = 0.3171).

We investigated viral dissemination across maternal organ systems and observed an increase in viral genome copy number in animals with prolonged or elevated viremia, specifically TH3, TH2, and PR2. At necropsy (40 dpi), up to 76 maternal tissues were collected and screened for ZIKV RNA (vRNA) per animal (see Table 1; full list in Table S2). Detected vRNA spanned multiple systems—including lymphatic, digestive, musculoskeletal, endocrine, reproductive, and both central and peripheral nervous systems—though distribution varied by individual. Consistent with previous work, viral loads were highest in lymph tissues, particularly axillary, inguinal, and mesenteric lymph nodes (LN) (up to 10^6^ copies of vRNA per µg of RNA) [38,63]. Notably, viral RNA was also detected in peripheral nerves in PR2 and PR3 (sciatic, femoral, and trigeminal nerves and brachial plexus), and in PR2, vRNA was detected within the brain stem, highlighting the neurotropic nature of ZIKV-PR in NHPs. While these findings align with prior reports of ZIKV crossing the blood–brain barrier in nonhuman primates, this is rarely observed in adult humans [63]. These nervous system findings were not observed in the ZIKV-TH dams. To assess potential strain-specific differences in tissue tropism, we compared pooled vRNA levels across the most frequently positive tissues. However, due to variability between animals, no statistically significant differences were found between the ZIKV-TH and ZIKV-PR groups (Figure S2A–C).

In addition, RNA was detected in tissue from two of three fetuses in each cohort at the study endpoint. Viral RNA-positive tissues (up to 10^4^ genomes) included brachial plexus, inguinal lymph node, and duodenum in the ZIKV-PR fetuses compared with ZIKV-TH fetuses, which had vRNA detected in frontal and occipital lobes, hamstring, and colon (Table 1). No vRNA was detected in the amniotic fluid or CSF of any of the fetuses in samples taken at necropsy. Combined, these data indicate that two of three fetuses from each group were infected following subcutaneous inoculation of the dams and that viral RNA persisted up to 40 dpi.

### 3.3. Neutralizing Antibodies Correlate with Decreased Viral Burden

To understand the impact of the humoral response on controlling viremia and viral dissemination to the developing fetus, we quantified the levels of ZIKV-positive IgM, IgG, and neutralizing antibody responses. Virus-specific IgM and IgG antibodies present as early as 5 dpi and 7 dpi, respectively, in both humans and rhesus macaques [64,65]. IgG antibodies are the main form of protection that crosses the placental barrier, and ZIKV-specific neutralizing antibodies have been found in the amniotic fluid, which is considered an important way that the body neutralizes viruses to protect the fetus from adverse outcomes [38,66]. Consistent with previous studies, we detected ZIKV-specific IgM as well as IgG antibodies as early as 3 dpi and 7 dpi, respectively, in all six infected macaques (Figure 2C). The IgM response rapidly rose, peaking at 14 dpi with the IgG levels peaking between 14 and 28 dpi and remaining elevated through 40 dpi. ZIKV-neutralizing antibodies were detected as early as 7 dpi using a focus reduction neutralization assay (Figure 2E). These neutralizing antibody levels reached 50% focus reduction neutralization titers (FRNT50) equal to plasma dilutions of 3.5 × 10^5^–4.6 × 10^6^ by 14 dpi (Figure 2E). Animals that developed the highest neutralizing antibody responses earlier by day 7, show less viral dissemination to maternal tissues (PR1, TH1) regardless of strain (Figure 1B, Table 1). Animals in which the neutralizing responses developed more slowly, by 10 dpi, had increased viral burden with PR2 (14%+), PR3 (16%+), and TH3 (10%+) with tissues ranging from 10^4^–10^6^ genomes per µg of tissue (Table 1). No significant differences in production of binding or neutralizing antibody responses were detected between the ZIKV infection groups.

### 3.4. Cellular Innate Immune Signatures Following ZIKV Infection

**Figure 3:**
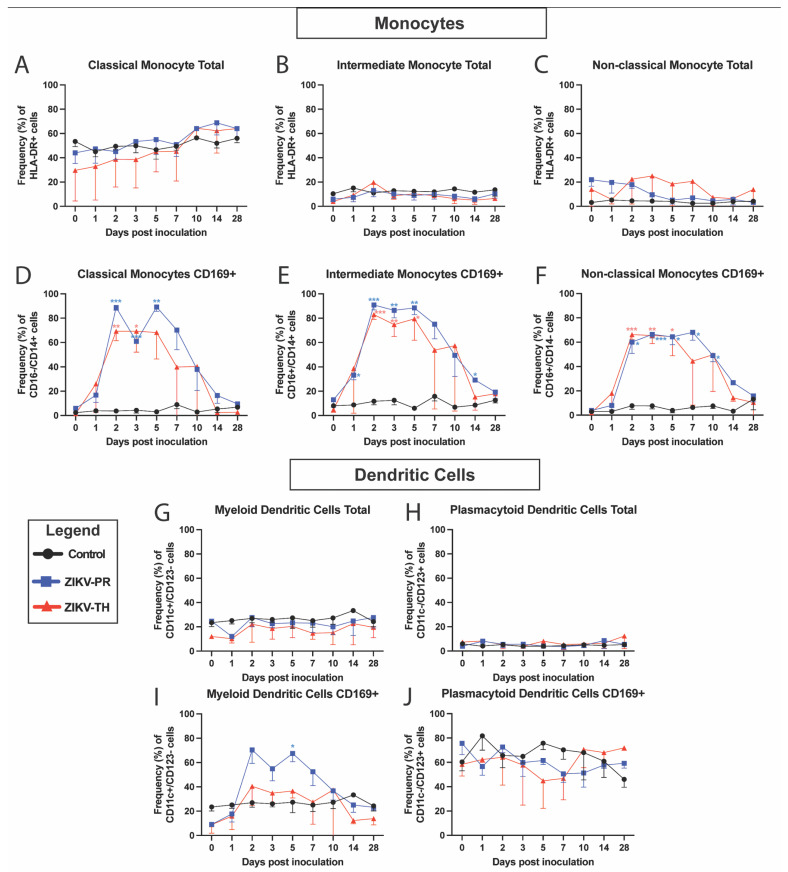
Longitudinal peripheral blood innate immune cell phenotype and activation. Rhesus macaque PBMC isolated at the indicated time points were stained with antibodies directed against cellular markers and analyzed for cell phenotype using flow cytometry. Changes in the longitudinal frequency of both total (**A**–**C**) and activated CD169+ (**D**–**F**) for classical monocytes, non-classical monocytes, and intermediate monocytes were quantified. Dendritic cells were separated into myeloid dendritic cells, and plasmacytoid dendritic cells were quantified as total (**G**,**H**) and activated CD169+ (**I**,**J**). Lines represent mean frequencies of the three animals for Control (black), ZIKV-PR (blue), ZIKV-TH (red), and error bars represent the standard error of the mean. Longitudinal changes in total or activated (CD169+) cells in the peripheral blood were analyzed using a mixed model approach with a Greenhouse–Geisser correction followed by Tukey’s multiple comparisons of control animals to ZIKV infected for each day post inoculation; for this analysis, only significant comparisons are shown: *** *p* = 0.0001, ** *p* < 0.001, and * *p* < 0.05.

Mouse studies have shown that innate immune responses, particularly interferon responses initiated by macrophages and dendritic cells, are crucial to clearing ZIKV infection [67,68]. To better understand if differences exist in the innate responses between the two viral strains, we monitored the development of the maternal immune response post-ZIKV inoculation by utilizing PBMCs isolated from blood samples at indicated time points. PBMCs were phenotypically analyzed by flow cytometry using antibody panels designed to identify innate immune cell activation (CD169+) in longitudinal samples (Figure S3). Monocyte populations, including classical (Figure 3D), intermediate (Figure 3E), and non-classical monocytes (Figure 3F), were highly activated with levels peaking at 2 dpi and remaining elevated until 5 dpi, which corresponds with the duration of viremia. These responses return to baseline levels by 28 dpi. Minimal change was observed in the frequencies of the monocyte subtype populations (Figure 3A–C). For the ZIKV-PR animals, activation in classical (*p* < 0.001), intermediate (*p* < 0.001), and non-classical (*p* < 0.001) monocytes was statistically significant compared with controls at indicated timepoints when analyzed using a mixed model approach with a Greenhouse–Geisser correction followed by Tukey’s multiple comparisons. Similarly, the ZIKV-TH group showed statistical significance in classical (*p* < 0.01), intermediate (*p* < 0.001), and non-classical (*p* < 0.001) monocytes. No statistical significance was observed between ZIKV strains at any time point.

Myeloid dendritic cells were also activated (CD169) in ZIKV-PR with a peak at 2 dpi before returning to baseline by 10 dpi (Figure 3G,I). Plasmacytoid dendritic cells showed no changes in the activation status. ZIKV-TH animals had no activation in either group. There were no significant changes to the total frequencies of either dendritic cell population for any cohorts (Figure 3H,J).

Adaptive immunity to flaviviruses mainly consists of neutralizing antibody responses, although the importance of CD4+ and CD8+ T cells in controlling CNS damage has been demonstrated in mice [69]. In humans, T cells have been shown to acquire the effector function and respond to pooled ZIKV peptides, with the most robust responses detected against capsid and envelope [70,71]. NHP studies have shown minimal T cell proliferation peaking around 10 dpi [37,38] and weak responses upon rechallenge, suggesting a paucity of cellular immunity [72]. To determine whether differences in cellular immunity exist between the strains, flow cytometry was performed using a panel of antibodies that distinguish the frequencies, phenotypes, and activation status of each of the CD4+ and CD8+ T cell subsets (Figures S4–S6). Central memory and Naïve CD4+ T cell proliferation (Ki67) remained unchanged; however, CD4+ effector memory (TEM) proliferation peaked at 10 dpi in some animals (Figure S5). This trend was observed to be higher in the ZIKV-TH, although not statistically significant (Figure S5C). There was also a steady expansion of granzyme B positive TEM CD4+ T cells in the ZIKV-PR animals and expansion in all three subgroups in the ZIKV-TH animals (Figure S5E,F). Proliferation of TCM CD8+ T cell populations increased over time, peaking at 7 dpi in ZIKV-PR cohorts, with no change seen within the ZIKV-TH cohorts (Figure S6B,C). However, these trends were not statistically significant when compared to controls (Figure S6A). Taken together, the peripheral T-cell responses remain minimal and similar between strains, although we cannot rule out the ability of these populations to respond to ZIKV-specific peptides.

### 3.5. Fetal Growth Dynamics

**Figure 4:**
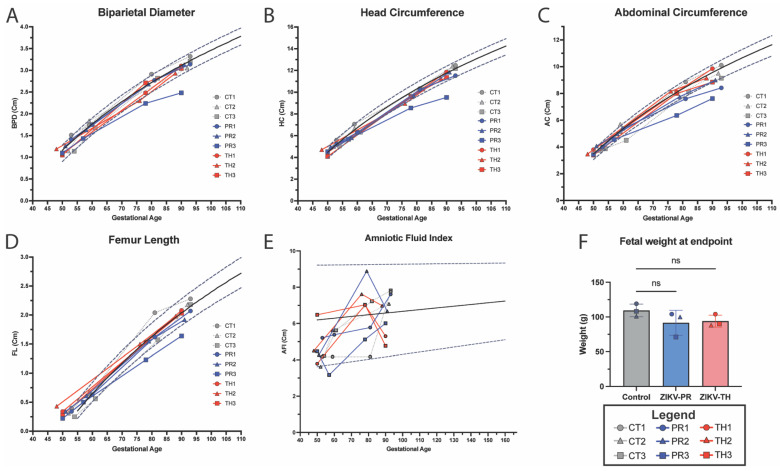
Fetal growth measured through ultrasound, amniotic fluid index, and fetal weight. Longitudinal ultrasound measurements of fetal growth across gestation including (**A**) Biparietal Diameter (BPD), (**B**) Head Circumference (HC), (**C**) Abdominal Circumference, and (**D**) Femur length in the six ZIKV-infected fetuses with control animals (*n* = 3) in gray, ZIKV-PR (*n* = 3) in blue, and ZIKV-TH (*n* = 3) in red plotted against historical controls, from ONPRC published data used to calculate the logarithmic regression for 50th percentile (black solid line) and for 10th and 90th percentiles, respectively (dashed lines). (**E**) Amniotic Fluid Index data were obtained by standard measurement of four quadrants and plotted against historical control with the logarithmic regression representing for 50th percentile (black solid line) and for 5th and 95th percentiles, respectively (dashed lines). (**F**) Fetal weight at necropsy (~GD 90) was measured in grams and analyzed using a one-way ANOVA (ns = *p* > 0.05) in GraphPad Prism v10.2.

The fetus and placenta of the pregnant animals were imaged using ultrasound to detect early signs of microcephaly, growth restriction, and to monitor blood flow dynamics. Fetal biometric measurements, including biparietal diameter, abdominal circumference, head circumference, and head diameter, were similar in fetuses from both ZIKV strains, with no statistically significant differences observed. Despite having confirmed vRNA detection in the central nervous tissue of the ZIKV-TH cohort, the animals had similar fetal biometric measurements to controls. Animal TH2 did appear to trend below the 10% threshold of historic controls at GD77 for biparietal diameter (Figure 4A) and head circumference (Figure 4B); however, this was recovered to normal measurements by GD90. Consistent with prior Asian-lineage NHP studies, ZIKV fetal infection is sporadic, and manifestations of disease are similar to human data at about 26% [30,73], indicating this cohort size could have been a factor. However, when compared to the ZIKV-PR cohort, the cohort on average fell below the median for its gestational age for head circumference (Figure 4B), abdominal circumference (Figure 4C), and femur length (Figure 4D), which was significantly decreased (*p* = 0.0317) compared to controls. Of note, PR3 was well below the top 10% of the linear regression line calculated from historical ONPRC control pregnancy biometry data [36], indicating potential growth restriction for this fetus.

To determine the role of amniotic fluid and growth dynamics, we calculated the amniotic fluid index using the standard four-quadrant measurements [36,37,38]. No significant changes outside the range of historical controls were observed in the ZIKV-TH cohort. However, when compared to the ZIKV-PR infected at 7 dpi, PR3 was below the lowest 5% of control animals for its gestational time point and remained below the median until necropsy. AFI is a marker of placental health and is often correlated with fetal growth, a value less than 5 cm is diagnostic in humans for oligohydramnios [74]. At necropsy, there were no significant differences in fetal weight when comparing the ZIKV-TH to the control group or when compared to the ZIKV-PR. The TH2 animal did have the lowest weight in the group, which is in line with the fetal measurements observed via ultrasound. When compared to the ZIKV-PR group, the PR3 fetus was smaller relative to controls, which is consistent with the observed lower estimated fetal weight measurements by ultrasound throughout the pregnancy (Figure 4F). It is important to note that maternal body weight dropped at 7 dpi in PR3, which may have contributed to the lower fetal growth observed in this case. However, it remains unclear whether this outcome was due to direct fetal infection, indirect effects of maternal illness, or other pregnancy-related complications, highlighting the complexity of factors influencing fetal outcomes during ZIKV infection. Together, these data suggest the presence of placenta dysfunction in animals within the ZIKV-PR cohort though variable between the animals.

### 3.6. Placental Pathology Aligns with Maternal Viremia and Viral Dissemination Within the Placenta

**Figure 5:**
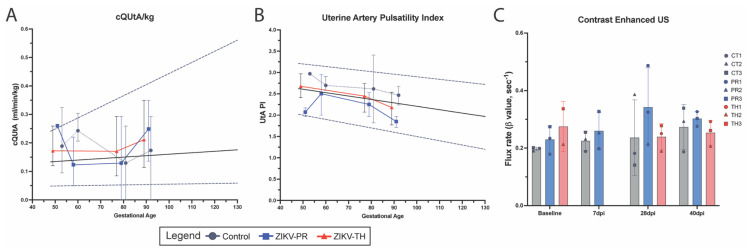
Uteroplacental hemodynamics. Uterine artery (UtA) Doppler measurements and calculations were performed using standard practices. Shown is the linear regression for the 50th percentile (solid line) and for 5th and 95th percentiles, respectively (dashed lines), based upon ONPRC historical data. (**A**) UtA blood flow (cQtA/kg) corrected for maternal body weight was calculated using the UtA diameter, maternal UtA cross-sectional area and volume of blood flow through the maternal UtA. (**B**) Doppler measurements were also used to calculate the UtA Pulsatility Index (PI). Graphs show averaged measurements and gestational ages for control animals (gray), ZIKV-PR (blue), and ZIKV-TH (red). (**C**) Contrast-enhanced ultrasound (CE-US) was used to measure the microvascular flux rate from control, ZIKV-PR, and ZIKV-TH groups, stratified across days post-infection.

To validate the fetal biometry data, we assessed uteroplacental hemodynamics for the placenta by Doppler ultrasound prior to infection and across gestation until 40 days post-infection (GD90). The calculated blood flow volume in the uterine artery (cQuta) corrected for maternal body weight demonstrated some variability between ZIKV animals but revealed an overall trend to increase with advancing gestational age (Figure 5A). The uterine artery pulsatility index (PI) did not deviate from control in any of the ZIKV animals (Figure 5B). Contrast-enhanced ultrasound (CEUS) was used to locate maternal spiral artery sources supplying individual placental cotyledons and measure blood flow dynamics (Figure 5C). While we did not observe any statistical difference between ZIKV strains, the flux rate constant (*β*) in TH3, an animal that had significantly elevated plasma viremia, was elevated. Similarly, PR3 remained higher than controls, which had elevated vRNA detection in disseminated tissues. The flux rate corresponds to increased blood flow velocity out of the maternal spiral artery, which can result in shear stress damage to the fetal villous tree. This also resulted in infarction, which was evident both grossly on examination and microscopically on histological analysis

**Figure 6:**
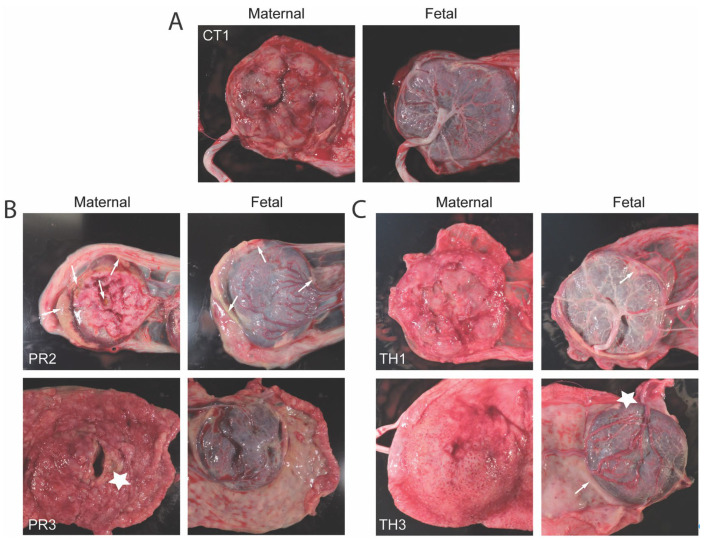
Gross placental pathology demonstrates infarction for ZIKV infection groups. (**A**) Control placenta at GD90 shown as a comparison of normal. (**B**) ZIKV-PR or (**C**) ZIKV-TH representative images of both maternal and fetal sides of the placenta. Milky-white appearance that is indicative of white blood cell infiltration is denoted by white arrows. Gross signs of infarction are indicated by white stars.

Although the uteroplacental hemodynamics were not significantly different between strains, placental characteristics were also monitored at necropsy. Many groups have shown placental pathology, including infarctions, decreased blood flow, and vasculitis induced by ZIKV-PR, but little is known about ZIKV-TH. On gross pathology, ZIKV-TH cohort had sporadic signs of infarction and white blood cell infiltration when compared to gestationally matched controls, shown by white arrows and stars, respectively (Figure 6). This was more prominent in the ZIKV-PR cohort, which is consistent with our previous work [38]. Following necropsy, the placental cotyledons or perfusion domains were mapped for ZIKV positivity using qPCR on 3–5 pooled villous samples. All the animals that had detectable placental viral RNA also all had higher levels of the virus detected in maternal tissues (Table 2). This finding highlights that both strains are associated with widespread dissemination and fetal transmission.

**Figure 7:**
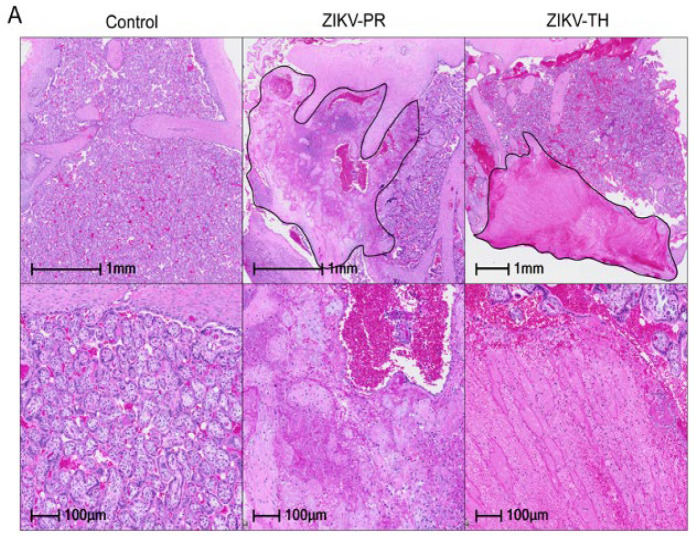

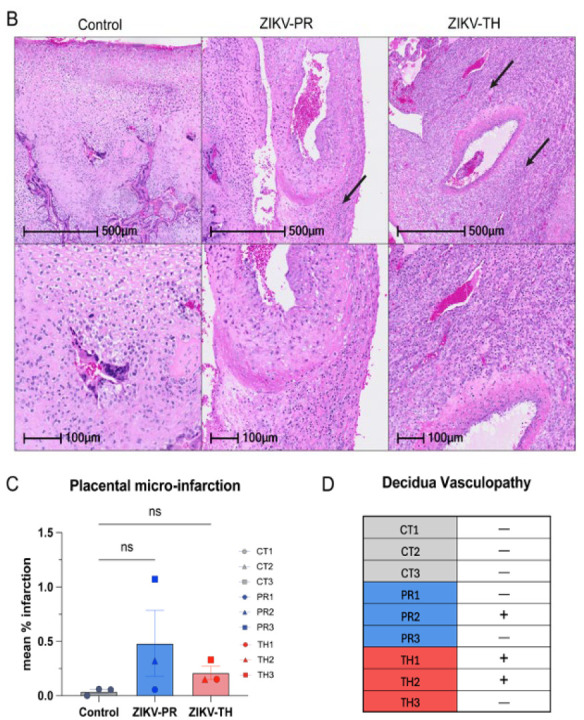
Placental histopathology of ZIKV-infected cases compared with gestational-age-matched negative controls. (**A**) Representative hematoxylin and eosin (H&E)–stained placental sections show infarctions (outlined in black) at low (top) and high (bottom) magnification. All six ZIKV-infected pregnancies exhibited infarctions in at least one cotyledon. All scale bars are shown in black with the top row at 1 mm and the bottom at 100 µm; original images were acquired at 20× magnification. (**B**) Representative hematoxylin and eosin (H&E) also showed chronic deciduitis with plasma cells and lymphoplasmacytic leukocytoclastic vasculitis (black arrows) in ZIKV-infected cases. Histological assessment of infarction was performed on 12–20 full-thickness placental sections collected from each cotyledon across both lobes of the placenta. Scale bars are shown in black with the top row at 500 µm and the bottom at 100 µm; original images were acquired at 20× magnification. (**C**) The percentage of infarcted sections per animal was quantified by a board-certified pathologist blinded to treatment group. Error bars represent standard deviation between animals (*n* = 3 per group). Statistical analysis was performed using a one-way ANOVA in Prism; ns = not significant (*p* > 0.05). (**D**) Presence (+) or absence (–) of decidual vasculopathy in at least one cotyledon is indicated for each case. All scale bars are shown in black; original images were acquired at 20× magnification.

Once mapped, two sections from each cotyledon were formalin-fixed and paraffin-embedded before staining with H&E. Slides were analyzed for signs of infarction, vasculitis, and additional pathology. Compared with sham negative controls, a three-fold increase in infarction frequency was observed in the ZIKV-PR inoculated group, which supports the gross pathology images (Figure 7A,C). Control animals exhibited little to no signs of infarction on gross pathology or histology. Decidual spiral artery leukocytoclastic vasculitis with evidence of spiral artery remodeling was identified in one of ZIKV-PR cases and two of ZIKV-TH cases (Figure 7B,D). Taken together with our previous findings, which suggested ZIKV-PR’s potential to induce placental damage and impact fetal outcomes, this study provides further evidence supporting the strain’s association with placenta damage.

## 4. Discussion

To investigate the pathogenic potential of the ZIKV-TH strain, we infected three rhesus macaques with ZIKV-TH and compared them with animals infected with the 2015 isolate from Puerto Rico, which we and others have previously demonstrated to induce placental damage [37,38,71].This comparative analysis focused on maternal, fetal, and placental outcomes to determine whether the observed differences in reported congenital ZIKV outcomes [6,13]—such as the higher incidence of CZS cases in the Americas compared to Southeast Asia—could be partially attributed to strain-specific pathogenesis, in addition to differences in surveillance or diagnostic capacity [75].

ZIKV-PR and ZIKV-TH exhibit similar kinetics of viremia with rapid plasma viremia, peaking between 1 and 3 dpi and resolving by 14 dpi with sporadic detection at later times. Despite similar viral loads, some clinical manifestations diverged. ZIKV-PR-infected animals showed transient weight loss and delayed weight gain during pregnancy (PR1, PR2), whereas ZIKV-TH-infected animals maintained a more stable maternal weight trajectory. Notably, viral RNA was broadly detected across maternal tissues in both groups, including neurotropic dissemination to the central and peripheral nervous systems for the ZIKV-PR (PR2 and PR3). These findings align with previous studies suggesting that maternal viremia correlates with systemic viral dissemination and the potential for fetal infection. However, no significant differences were observed in the magnitude or distribution of viral RNA between the two strains, suggesting that both viruses are capable of systemic dissemination with comparable efficiency.

Placental pathology and fetal outcomes provided further insight into strain-specific impacts. ZIKV-PR was associated with decreased estimated fetal weight in utero for animals PR1 and PR3, including decreased head circumference, abdominal circumference, and femur length measurements, as well as lower fetal weight at necropsy, suggestive of placental dysfunction. However, it is important to note that the changes in maternal weight also likely contributed to the decreased fetal growth observed. Additionally, it is unclear whether the observed decrease in fetal growth would have persisted or resolved later in gestation, as pregnancies were not carried to full term. Moreover, gross and histological analyses of placental tissue revealed higher frequencies of infarctions and leukocytoclastic vasculitis in ZIKV-PR-infected animals, consistent with changes associated with impaired placental function. In contrast, while ZIKV-TH infection showed some evidence of placental remodeling, it did not result in slowed fetal growth. This finding suggests that while ZIKV-TH retains neurotropic and placental invasive capabilities, in this cohort, we observed less placental damage, which may contribute to the lower rate of congenital ZIKV syndrome seen within the region. However, with our small cohort size, it is difficult to determine whether these differences are strain-specific or reflective of individual variability.

When compared to other ZIKV strains studied in non-human primate models, ZIKV-TH appears to follow a trajectory that is similar to other Asian-lineage viruses rather than African-lineage strains, which have demonstrated more severe and rapid pregnancy loss. For example, the African lineage strain Dakar 41524 has been associated with high-dose-dependent fetal demise, with infection reaching the maternal–fetal interface (MFI) within six days and resulting in pregnancy loss in a subset of infected animals [76,77,78,79,80]. In contrast, Asian-lineage strains—including PRVABC59 and Cambodia 2010—demonstrate a spectrum of outcomes ranging from fetal neurodevelopmental abnormalities to increased immune activation in maternal tissues without immediate fetal loss (Table S2) [22]. The ability of ZIKV-TH to persist in reproductive tissues without significant pregnancy loss suggests that it may induce a more prolonged placental infection, potentially mirroring the pathogenic mechanisms observed in Asian-lineage strains rather than the acute pathology that is seen with African-lineage isolates. However, gestational timing of infection does appear to play a role in the acute pathogenesis.

The maternal immune response likely contributed to controlling viral dissemination and mitigating adverse outcomes. Consistent activation of innate immune cell populations—including classical, non-classical, and intermediate monocytes, myeloid dendritic cells, and NK cells—mirrored the kinetics of viremia, peaking between 2 and 4 dpi before returning to baseline by 14 dpi. While ZIKV-PR-infected animals exhibited slightly elevated and more sustained immune activation compared to ZIKV-TH-infected animals, this difference was largely driven by two ZIKV-TH-infected animals with lower viremia, which correlated with reduced activation of these immune cell populations. These subtle differences in immune activation may contribute to variability in disease severity between individual animals rather than reflecting a fundamental distinction between the viral strains.

Interestingly, despite the lower observed rates of placental pathology following ZIKV-TH infection compared to ZIKV-PR, the immune response to ZIKV-TH shares similarities with other studied ZIKV infections in NHPs (Table S2) [22]. Previous studies have shown robust maternal immune activation, including upregulation of pro-inflammatory pathways and monocyte activation [63,81,82,83]. While African-lineage strains such as Dakar 41524 demonstrate rapid maternal–fetal transmission, Brazilian and Puerto Rican strains have been associated with more prolonged immune responses that could contribute to sustained placental inflammation and pathology. The fact that ZIKV-TH elicits an immune response comparable in magnitude to these strains suggests that its relative attenuation in fetal outcomes may be driven by differences in placental resilience or localized immune regulation rather than a fundamental reduction in maternal immune activation. Further studies are needed to determine how host-specific factors, including genetic and immunological background, may shape disease severity in ZIKV-TH infections.

Both groups generated similar adaptive immune responses, with comparable kinetics of IgM and IgG ZIKV-binding antibodies and neutralizing antibody responses. Interestingly, at later time points there appeared to be an IgG elevated group of three animals, though this did not appear to correlate with any disease outcomes. Notably, animals that developed neutralizing antibodies earlier, by 7 dpi, exhibited reduced viral dissemination to maternal tissues regardless of the infecting strain. Moreover, animals with higher neutralizing antibody titers at 7 dpi had undetectable viremia, whereas those that developed neutralizing responses later (by 10 dpi) exhibited increased viral burden. These findings suggest that the timing and strength of the individual immune response, rather than strain-specific differences in immune evasion, were the primary drivers of disease outcomes. Importantly, no significant differences in the magnitude of binding or neutralizing antibody responses were detected between ZIKV-PR and ZIKV-TH, indicating that both strains elicit robust adaptive immunity with only subtle variations that may influence tissue-level viral control and pathogenesis.

## 5. Conclusions

In conclusion, this study demonstrates that while ZIKV-PR and ZIKV-TH share highly similar infection dynamics, immune responses, and maternal-fetal transmission potential, subtle differences in immune activation and placental pathology may influence the severity of disease outcomes. These findings suggest that host factors, including pre-existing immunity and individual variation in immune response kinetics, may play a more critical role in shaping ZIKV-associated pregnancy outcomes than inherent differences between these two viral strains. Future studies with larger cohorts are needed to further delineate the molecular and immunological factors that contribute to regional differences in congenital ZIKV manifestations and to explore potential therapeutic strategies to enhance maternal immune defenses against ZIKV infection.

## Figures and Tables

**Table 1:** ZIKV tissue distribution. ZIKV RNA levels in the tissues of animals were quantified either in duplicate or triplicate, using one-step qRT-PCR. ZIKV-PR dams (D) and fetuses (F) are indicated in blue columns and the ZIKV-TH cohort is in red columns. Total RNA was extracted using Trizol on precleared samples following bead beating. Approximately 76 different tissues were assessed for the presence of viral RNA in dams and 60 different tissues in the fetuses. Mean quantity values are presented as log10 viral copies per µg of RNA with undetectable samples indicated as —, and tissues not tested indicated in gray. Shown are the tissues with positive detection in at least one of the animals per cohort. The approximate limit of detection was 400 genomes/mL (2.6log10 genomes), based on a detection limit of 100 genomes per reaction, as determined in Supplemental Figure S1.

		ZIKV-PR						ZIKV-TH					
		PR1-D	PR1-F	PR2-D	PR2-F	PR3-D	PR3-F	TH1-D	TH1-F	TH2-D	TH2-F	TH3-D	TH3-F
	% vRNA+ tissues	3/62 (4.8%)	1/43 (2%)	10/63 (16%)	2/43 (4.7%)	9/63 (14%)	0/49 (0%)	3/67 (4.5%)	0/48 (0%)	6/63 (9.5%)	3/55 (5%)	5/49 (10%)	1/36 (2.7%)
Lymph tissue	Axillary LN	3.76	**—**	3.78	**—**	6.20	**—**	5.52	**—**	5.68	**—**		
	Inguinal LN	**—**	2.90	2.78	**—**	5.84	**—**	**—**	**—**	**—**	**—**	4.24	
	Mesenteric LN	**—**	**—**	3.66	**—**	5.72	**—**	4.49	**—**	4.07	**—**	**—**	**—**
	Thymus	**—**	**—**	**—**	**—**	**—**	**—**	**—**	**—**	**—**	**—**	3.31	**—**
Digestive system	Stomach	**—**	**—**	**—**	**—**	**—**	**—**	**—**	**—**	**—**	**—**	3.57	**—**
	Duodenum	**—**	**—**	**—**	2.99	**—**	**—**	**—**	**—**	**—**	**—**	**—**	
	Colon	**—**	**—**	**—**	**—**	**—**	**—**	**—**	**—**	**—**	**—**	**—**	4.34
Musculoskeletal	Biceps Brachii	**—**		**—**		**—**		**—**	**—**	3.18		**—**	**—**
	Elbow	**—**		3.49		**—**		**—**	**—**	**—**	**—**	**—**	
	Finger	**—**		3.55		5.54		4.63	**—**	3.77	**—**	**—**	
	Hand/Wrist	**—**	**—**	3.93	**—**	5.65	**—**	**—**		**—**	**—**	**—**	**—**
	Quadriceps muscle	**—**		**—**		**—**		**—**	**—**	2.91	**—**	3.03	**—**
	Hamstring	2.87		**—**		**—**		**—**	**—**	**—**	3.36	**—**	**—**
	Foot/Ankle	**—**	**—**	**—**	**—**	4.55	**—**	**—**		**—**	**—**	**—**	**—**
Genito-urinary	Uterus	**—**	**—**	**—**	N/A	**—**	N/A	**—**	**—**	4.77	**—**	**—**	
	Vagina	**—**		**—**		**—**		**—**		**—**		3.11	
	Urethra	2.95	**—**	**—**	**—**	**—**	**—**	**—**	**—**	**—**	**—**	**—**	
Nervous tissue													
PNS	Brachial Plexus	**—**	**—**	**—**	3.00	**—**	**—**	**—**	**—**	**—**	**—**	**—**	
	Trigeminal Nerve	**—**		3.47		5.10		**—**		**—**			
	Femoral Nerve	**—**		2.90		**—**		**—**		**—**			
	Sciatic Nerve			2.73		5.03	**—**	**—**	**—**		**—**	**—**	
CNS	Pituitary Gland	**—**	**—**	**—**	**—**	**—**	**—**	**—**		**—**	**—**	**—**	
	Frontal Lobe	**—**		**—**		**—**		**—**	**—**	**—**	3.71	**—**	
	Occipital lobe	**—**		**—**		**—**	**—**	**—**		**—**	2.99	**—**	**—**
	Brain Stem	**—**		2.61		**—**		**—**		**—**		**—**	
Endocrine	Arendal Glands	**—**	**—**	**—**	**—**	4.76	**—**	**—**	**—**	**—**	**—**	**—**	**—**

**Table 2:** Placental viral loads. Total RNA was isolated from mapped placental cotyledons from both primary (1°) and secondary (2°) discs. All available cotyledons were tested; however, the number of cotyledons varied between placentas. Gray shading indicates cotyledons that were absent in respective animals. ZIKV RNA levels were quantified using a one-step-qRT-PCR assay in either duplicate or triplicate for ZIKV-PR (blue) and ZIKV-TH (red). Mean quantity values based on standard ZIKV FFU are presented as log10 viral copies per µg of RNA. A red intensity gradient is used to indicate vRNA levels, with dark red representing higher viral RNA quantities and lighter shades indicating lower values. The limit of detection was approximately 400 genomes (2.6log10) per µg of total RNA.

	ZIKV-PR						ZIKV-TH					
	PR1		PR2		PR3		TH1		TH2		TH3	
Placental Disc	1°	2°	1°	2°	1°	2°	1°	2°	1°	2°	1°	2°
Cotyledon #												
1	—	—	3.58	—	—	—	—	—	—	—	—	3.58
2	—	—	—	—	—	—	—	—	—	—	3.64	—
3	—	—	1.74	2.72	—	—	—	—	—	—	5.22	5.16
4	—	—	—	—	—	—	—	—	—	—	—	3.58
5	1.07	—	—	—	—	—	—	—	—			5.90
6	—	—	2.08	2.19	—	—	—	—	2.56			3.87
7	—	—	—	2.10	—	3.29	—	—	—			
8	—	—	—	—			—	—	—			4.15
9	—		—	1.93			—	—				5.04
10	—		2.87				—					
11	—		2.09				—					
12	—											

## Data Availability

The original contributions presented in the study are included in the article/Supplementary Materials, further inquiries can be directed to the corresponding author.

## Supplementary Materials

The following supporting information can be downloaded at: https://www.mdpi.com/article/10.3390/v17060762/s1, Table S1: Nonhuman primate pregnancy models of in utero Zika virus infection; Table S2: Complete tissue list tested for ZIKV dissemination; Figure S1: Validation of RT-PCR quantification of ZIKV (PRVABC59 and MU1-2017) vRNA; Figure S2: ZIKV tissue distribution; Figure S3: Flow cytometry gating strategy for natural killer cells/monocytes/dendritic cell panel; Figure S4: Flow cytometry gating strategy for T-cell panel; Figure S5: Peripheral Blood CD4 T cell proliferation and granzyme B expression following ZIKV inoculation; Figure S6: Peripheral Blood CD8 T cell proliferation and granzyme B expression following ZIKV inoculation.
