# Supplementary material for: Comparative Analysis of Two Zika Virus Isolates in a Rhesus Macaque Pregnancy Model
Source: Viruses. 2025 May 27;17(6):762. doi: 10.3390/v17060762 (PMC12197658; doi:10.3390/v17060762)
Supplement: Supplementary file 1 [file viruses-17-00762-s001.zip › viruses-3609710-supplementary.pdf]

Supplementary Materials:

Table S1

Non-Human Primate	Strains used	Outcomes of studies	Citation
Macaca mulatta Rhesus Macaque	PeurtoRican_2015_PRVABC59	Subcutaneous, Higher ZIKV-specific antibody titers in dams with worse virologic control could be used as a biomarker of poor maternal control of infection	[23]
Macaca mulatta Rhesus Macaque	A.africanus-tc_Senegal_1984_DAKAR_41524	Subcutaneous, high-dose exposure to African-lineage ZIKV causes pregnancy loss in 3/8 pregnancies (GD45)	[78]
Macaca mulatta Rhesus Macaque	A.africanus-tc_Senegal_1984_DAKAR_41524	Intravaginal, at approximately 45 days gestation (17-18 days post-challenge), two of the three dams were found with nonviable embryos by ultrasound. Viral RNA was detected in recovered tissues and at the maternal-fetal interface (MFI) in both cases	[80]
Macaca mulatta Rhesus Macaque	A.africanus-tc_Senegal_1984_DAKAR_41524	ZIKV can reach the maternal fetal interface by six days after infection and infect the fetus by ten days and infection in fetal maternal interface	[77]
Macaca mulatta Rhesus Macaque	A.africanus-tc_Senegal_1984_DAKAR_41524	Subcutaneous, viral RNA in fetus, early fetal demise within 20 dpi.	[41]
Macaca mulatta Rhesus Macaque	PeurtoRican_2015_PRVABC59	Subcutaneous, 26% experienced fetal demise later in pregnancy with few clinical signs of infection	[37]
Macaca mulatta Rhesus Macaque	PeurtoRican_2015_PRVABC59	Subcutaneous Viral RNA in fetus, increased maternal-placental-fetal inflammatory response, placenta damage, reduced oxygen permeability of the placental villi	[38]
Macaca mulatta Rhesus Macaque	ZIKV-BR	Subcutaneous Fetal neuropathology, neuroprogenitor apoptosis, placental pathology	[40]
Macaca mulatta Rhesus macaque	Brazil_SPH2015	Intra-amniotic Viral RNA in fetal and placental tissues, early death of fetus, fetal ZIKV neurotropism, neuropathology at the end of gestation	[24]
Macaca mulatta Rhesus macaque	Brazil_SPH2015	Intravaginal, Viral RNA in female reproductive tract, increased cytokine levels in cervicovaginal lavage	[29]
Macaca mulatta Rhesus macaque	PeurtoRican_2015_PRVABC59, Brazil_2015_ BeH815744	Subcutaneous, Virus in lymph nodes in both paracortical regions, germinal centers, and cerebrospinal fluid, upregulation of proinflammatory and anti-apoptotic signaling pathways	[63]
Macaca mulatta Rhesus macaque	ZIKV-Brazil_HS-2015-BA-01 clinical isolate	Intravenous, Activation of lymphoid tissues	[82]
Macaca mulatta Rhesus macaque	PeurtoRican_2015_PRVABC59	Subcutaneous, Virus in multiple lymphoid tissues, viral RNA in lymphoid and joint/muscle tissues	[49]

Macaca nemestrina Pigtail macaque	Brazil_2015_MG	Subcutaneous, Increase of innate immune cells in blood, lymph nodes, and mucosal tissues, activation of monocytes in peripheral lymph nodes	[81]
Macaca nemestrina Pigtail macaque	Cambodia_2010_FSS13025<br>Brazil_2015_MG	Subcutaneous Decreased fetal non-cortical brain volume, injury in ependymal epithelium with underlying gliosis, reduced late fetal neuronal progenitor cells in the subventricular zone	[25]
Callithrix jacchus<br>Marmoset	Brazil_SPH2015	Intramuscular Fetal demise and abortion, increase of proinflammatory cytokines, fetal neurocellular disorganization, viral RNA in placenta and fetus	[42]
Macaca mulatta<br>Rhesus macaque,<br>*Macaca fascicularis*<br>Cynomolgus macaque	Thai_2013_PLCal_ZV<br>PeurtoRican_2015_PRVABC59	Viral RNA in female reproductive tissues	[26]
Macaca fascicularis<br>Cynomolgus macaque	Human_1968_Nigeria_IBH30656<br>PeurtoRican_2015_PRVABC59,<br>Cambodia_2010_FSS13025	Subcutaneous, Virus in the testis	[27]
Papio Anubis<br>Olive baboon	Frenchpolynesian_H_PF_2013,<br>PeurtoRican_2015_PRVABC59	Vaginal deposition of ZIKV-infected semen, Viral RNA in lymph nodes	[28]

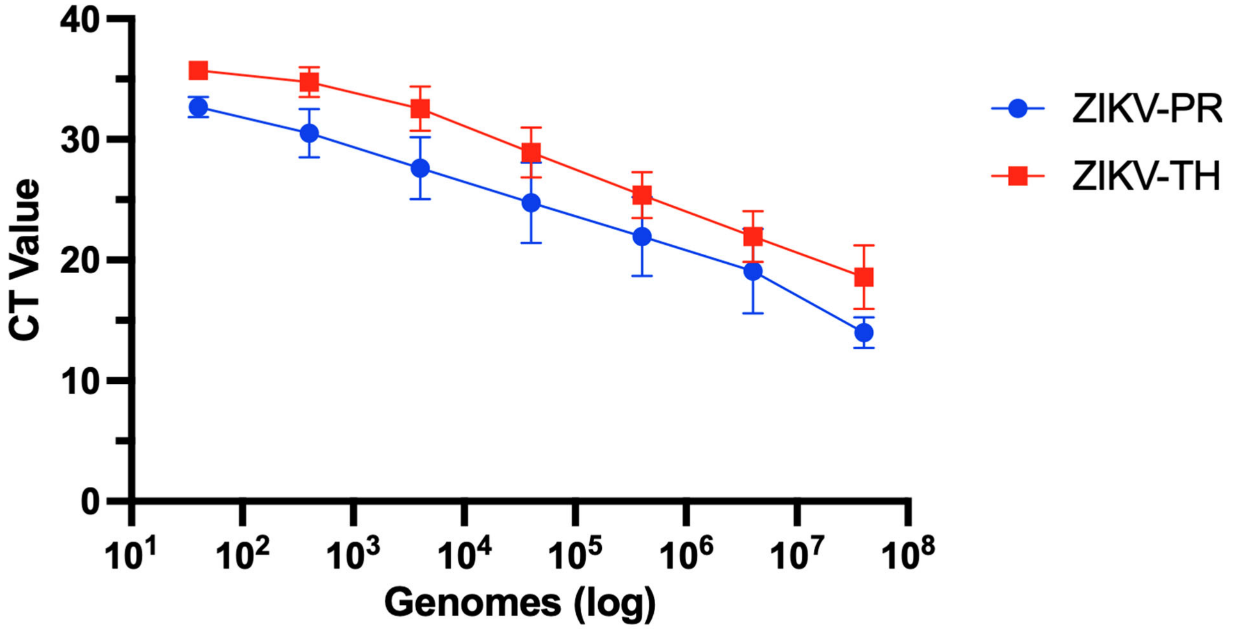

Figure S1. Validation of RT-PCR quantification of ZIKV (PRVABC59 and MU1-2017) vRNA. RNA was isolated from purified PRVABC59 (ZIKV-PR) or MU-127 (ZIKV-TH) tittered stocks. Total RNA was quantified via spectrophotometry and used to calculate genomes/ml. The stock was diluted to the indicated genome number and analyzed by one-step q-RT-PCR in triplicate using primers and conditions described in the methods. The amplification cycle threshold (CT) is plotted against total viral genomes for ZIKV-PR and ZIKV-TH for 11-16 replicates except for the lowest dilution with 3 replicates.

Table S2. Complete tissue list tested for ZIKV dissemination. ZIKV RNA levels in the tissues of animals were quantified either in duplicate or triplicate using one-step qRT-PCR. Total RNA was generated using Trizol on precleared samples following bead beating. Approximately 76 different tissues were assessed for the presence of viral RNA in dams and 60 different tissues in the fetus. Mean quantity values are presented as log10 viral copies per μg of RNA with undetectable samples indicated as —, and tissues not tested indicated in grey. Shown are the tissues with positive detection in at least one of the animals per cohort. The approximate limit of detection was 400 genomes/ml (2.6log10 genomes) or 100 genomes per reaction determined in supplemental figure 1. Red indicates above LOD and blue indicates below LOD with amplification detected.

		ZIKV-PR						ZIKV-TH					
		PR1-D	PR1-F	PR2-D	PR2-F	PR3-D	PR3-F	TH1-D	TH1-F	TH2-D	TH2-F	TH3-D	TH3-F
Lymph tissues	Cervical LN	—		1.25		—		—		—			
	Submandibular LN	1.48				—		—		—			
	Axillary LN	3.76	—	3.78	—	6.20	—	5.52	—	5.68	—		
	Inguinal LN	1.38	2.90	2.78	—	5.84	—	—	—	—	—	4.24	
	Mesenteric LN	2.40	—	3.66	1.42	5.72	—	4.49	—	4.07	—	—	—
	Retroperitoneal LN	—		—		—		—		—			
	Bone Marrow		1.79		—		—				—		

	Spleen	–	–	1.60	–	–	–	–	–	–	–		–
	Thymus	–	–	–	–	–	–	–	–	–	–	3.31	–
	Tonsil	1.91		–		–		–		–		–	
Circulatory system	Heart	2.00	–	–	–	2.16	–	–	–	–	–	–	–
	Lung	–	–	1.28	–	–	–	–	–	–	–	–	–
	Aorta							–	–				–
	Umbilical Cord		1.63		–		–				–		–
Digestive system	Parotid Gland	–	–	1.71	1.60	–	–	–	–	–	–	–	–
	Submandibular Gl.	1.72	–	–	–	–	–	–	–	–	–		–
	Esophagus	–	–	–	–	–	–	–	–	–	–	–	–
	Stomach	–	–	–	2.07	–	–	–	–	–	–	3.57	–
	Liver	–	–	–	–	–	–	–	–	–	–	–	–
	Gall Bladder	1.87	–	–	–	–	–	–	–	–	–	–	
	Pancreas	1.65	–	–	–	–	–	–	–	–	–	–	–
	Small Intestine												–
	Duodenum	–	–	–	2.99	–	–	–	–	–	–	–	
	Jejunum	–	–	–	–	–	–	–	–	–	–	–	
	Ileum	1.58	–	–	–	–	–	–	–	–	–	–	
	Cecum	–		–		–		–	–	–		–	–
	Colon	–	2.16	–	–	–	–	–	–	–	–	–	4.34
Musculoskeletal	Skin Upper Torso	1.71	–	1.57	–	–	–	–	–	–	–	–	–
	Upper Arm		–		–		–				–		–
	Triceps Brachii	2.13		–		–		–	–	–		–	–
	Biceps Brachii	1.67		–		–		–	–	3.18		–	–
	Lower arm		–		–		–				–		–
	Brachioradialis	1.61		2.11		–		–		–			
	Elbow	2.14		3.49		–		–	–	–	–	–	
	Finger	2.28		3.55		5.54		4.63	–	3.77	–	–	
	Hand/Wrist	–	–	3.93	–	5.65	–	–		–	–	–	–
	Upper Leg		–		–		–				–		–
	Quadriceps muscle	1.52		–		–		–	–	2.91	–	3.03	–
	Hamstring	2.87		1.91		–		–	–	–	3.36	–	–
	Lower Leg		1.60		–		–				–		–
	Knee	1.74	1.48	–	–	–	–	–		–	–		
	Soleus muscle	1.66		–	–	–	–	–	–	–	–	–	
	Foot/Ankle	–	1.61	1.94	–	4.55	–	–		–	–	–	–
	Toe	2.11		1.75		–		–		–		–	
Genito-urinary	Ovaries	–	–	–	–	–	–	–	–	–	–	–	–
	Fallopian Tube	–	2.21	–	N/A	–	–	–	–	–	–	–	
	Uterus	–	–	–	N/A	–	–	–	–	4.77	–	–	
	Cervix	2.02		1.99	N/A	–		–	–	–	–	–	

	Vagina	–		–		1.51		–		–		3.11	
	Prostate	N/A	N/A	N/A	–	N/A	–	N/A		N/A	–	N/A	
	Testes	N/A	N/A	N/A	2.32	N/A	–	N/A		N/A	–	N/A	
	Seminal Vesicles	N/A	N/A	N/A	1.94	N/A	–	N/A		N/A	–	N/A	
	Kidney	–	–	–	–	–	–	–	–	–	–	–	–
	Urinary Bladder	–	–	–		–	–	–	–	–	–	–	
	Ureter							–	–				
	Urethra	2.95	–	–	–	–	–	–	–	–	–	–	
Nervous tissue													
PNS	Brachial Plexus	–	–	–	3.00	–	–	–	–	–	–	–	
	Dorsal Root Ganglion	2.02		–		–		–		–			
	Trigeminal Nerve	–		3.47		5.10		–		–			
	Femoral Nerve	–		2.90		–		–		–			
	Sciatic Nerve			2.73		5.03	–	–	–		–	–	
CNS	Pituitary Gland	–	–	–	1.90	–	–	–		–	–	–	
	Frontal Lobe	–		–		–		–	–	–	3.71	–	
	Parietal Lobe	–		–		–		–		–			
	Temporal Lobe	–		–		–		–	–	–			–
	Hippocampus							–					–
	Basal Ganglion	0.95		1.77		–		–		–		–	
	Occipital lobe	–		–		–	–	–		–	2.99	–	–
	*Eyes	1.53	1.82	–	–	–	–	–	–	–	–	–	–
	Cerebellum	1.65		–		–		–	–	–		–	–
	Brain Stem	–		2.61		–		–		–		–	
	Spinal Cord												–
	Cervical Spinal Cord	–	–	2.11	–	–	–	–	–	–	–	–	
	Thoracic Spinal cord	–	–	–	1.85	–	–	–	–	–	–	–	
	Lumbar Spinal cord	–	–	–	1.82	–	–	–	–	–	–	–	
Endocrine	Adrenal Gland	–	2.00	–	2.48	4.76	–	–	–	–	–	–	–
	Mammary Gland	1.68	1.65	–	1.88	–	–	–	–	–	–		
	Thyroid Gland	1.83	–	–	2.51	–	–	–	–	–	–	–	

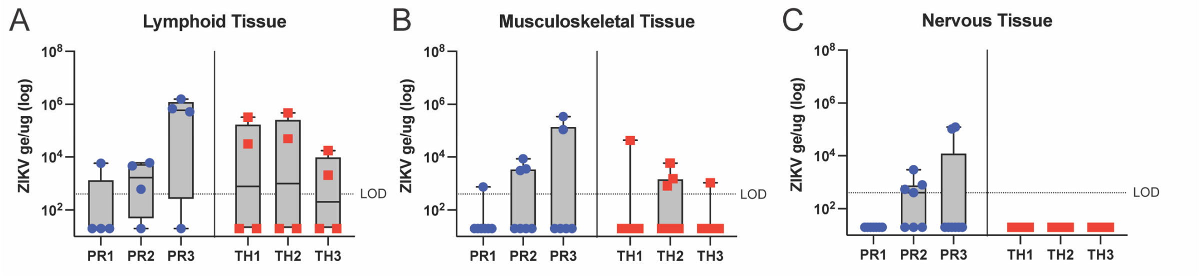

Figure S2. ZIKV tissue distribution. (A) Graphical depiction of viral loads from Table 1 were detected via one-step qRT-PCR. The limit of detection (LOD) was 400 copies ZIKV RNA per ml of tissue lysate with undetectable samples graphed as 50 copies vRNA/µg of tissue. Graphs represent tissues that had at least one animal with positive viral detection and are separated into the three tissue types that have the highest positive tissues (A) lymphoid, (B) Musculoskeletal, and (C) Nervous tissue. A Two-way ANOVA with a Greenhouse- Gaussian correction followed by Tukey's multiple comparisons for each tissue type from animals infected with ZIKV-PR and ZIKV-TH. For this analysis: not shown on graph is represented with ns, * represents samples with $p > 0.05$ for lymphoid and musculoskeletal and * $p < 0.05$ for nervous tissue.

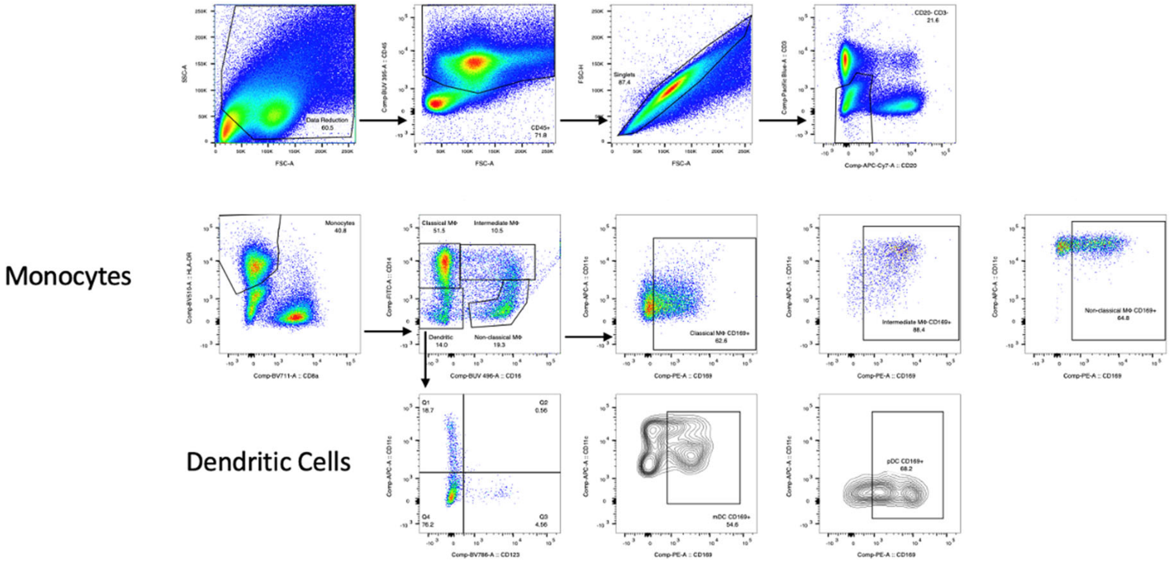

Figure S3. Flow cytometry gating strategy for natural killer cells/monocytes/dendritic cell panel. Natural killer cells were defined as CD3-/CD20- and lymphocyte-gated with CD56bright, CD56+/CD16+,

CD56dim/CD16+ separated populations. Monocytes and macrophages were defined as CD3-/CD20-/CD8-/HLA-DR+ with classical monocytes being CD16-/CD14+, intermediate monocytes being CD16+/CD14+, and non-classical monocytes being CD16+/CD14-. DCs were defined as CD3-/CD20-/CD8-/HLA-DR+/CD16-/CD14- with myeloid DCs being CD11c+/CD123- and plasmacytoid DCs being CD11c-/CD123+. Activated cells within each subset were defined as CD169+ set at D0 baseline for each animal.

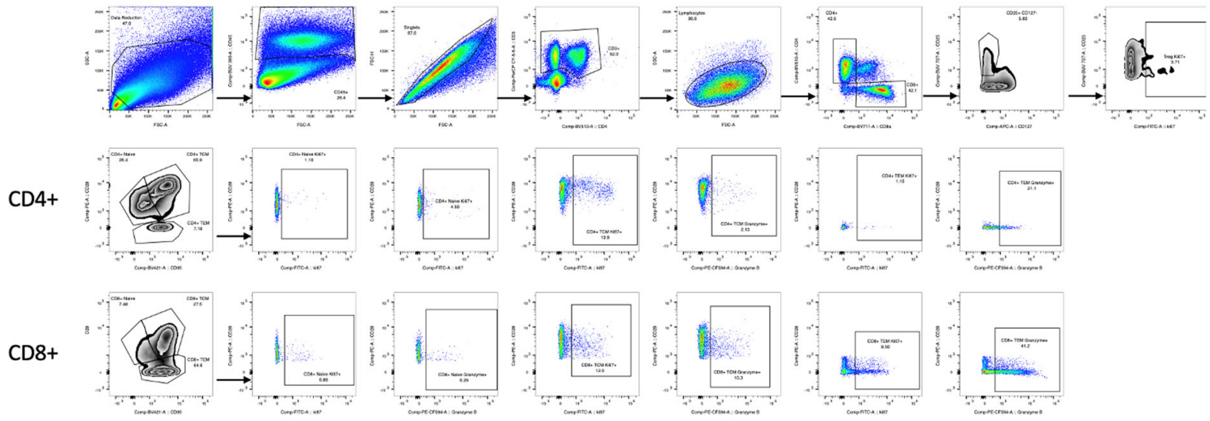

Figure S4. Flow cytometry gating strategy for T-cell panel. CD4+ or CD8+ T-cell subsets were defined as either naïve (CD28+/CD95-), central memory (CD28+/CD95+), and effector memory (CD28-/CD95). Proliferating (Ki67+) T-cells and granzyme B expressing (granzyme B+) T-cells within each subset were quantified using this gating scheme with D0 set as baseline for each animal.

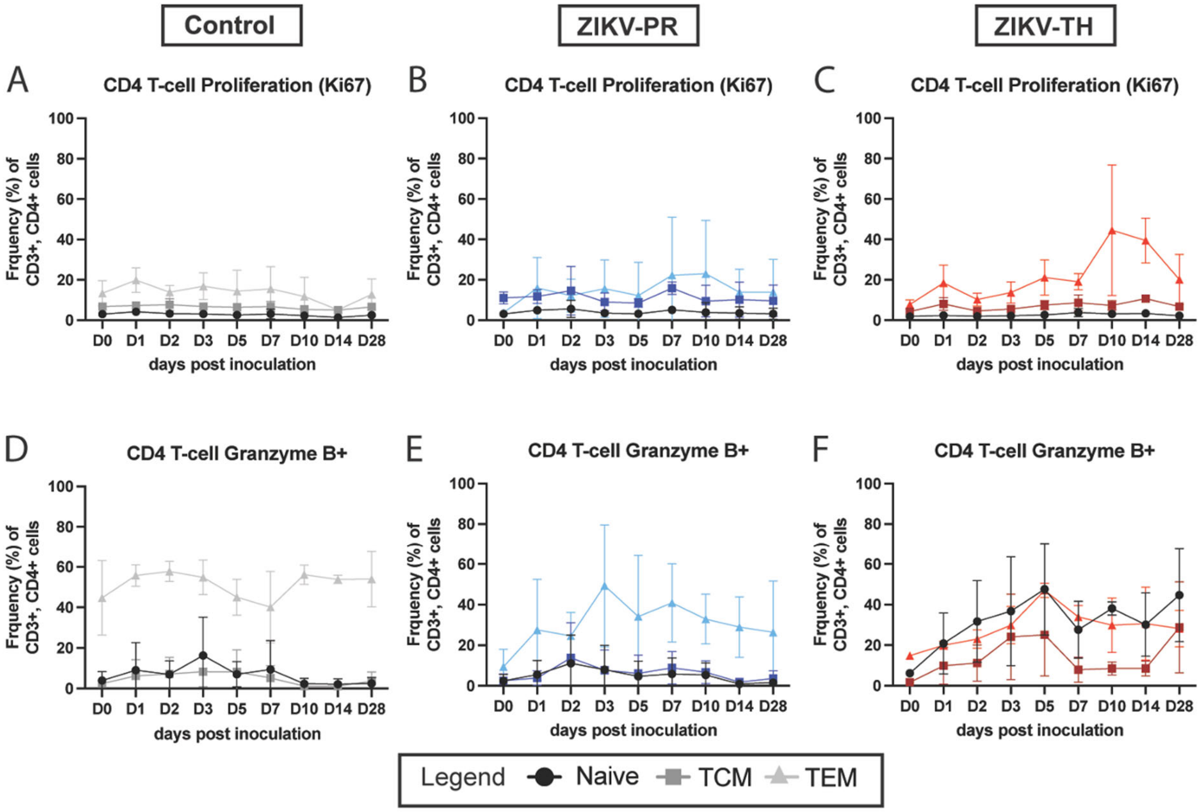

Figure S5. Peripheral Blood CD4 T cell proliferation and granzyme B expression following ZIKV inoculation. Rhesus macaque PBMC from indicated time points post-inoculation were analyzed for T cell phenotype using flow cytometry. Proliferating naïve, central memory, and effector memory CD4+ T-cell (A-C) proliferation and (D-F) granzyme B expression (granzyme B+) are shown for control, ZIKV-PR and ZIKV-TH groups. Longitudinal changes in proliferating (Ki67+) and activated (Granzyme B+) cells in the peripheral blood were analyzed using a mixed model approach with a Greenhouse–Geisser correction followed by Tukey's multiple comparisons of control animals to ZIKV infected for each day post-inoculation; for this analysis for this analysis all time points indicated ns $p > 0.05$ (not shown on figure).

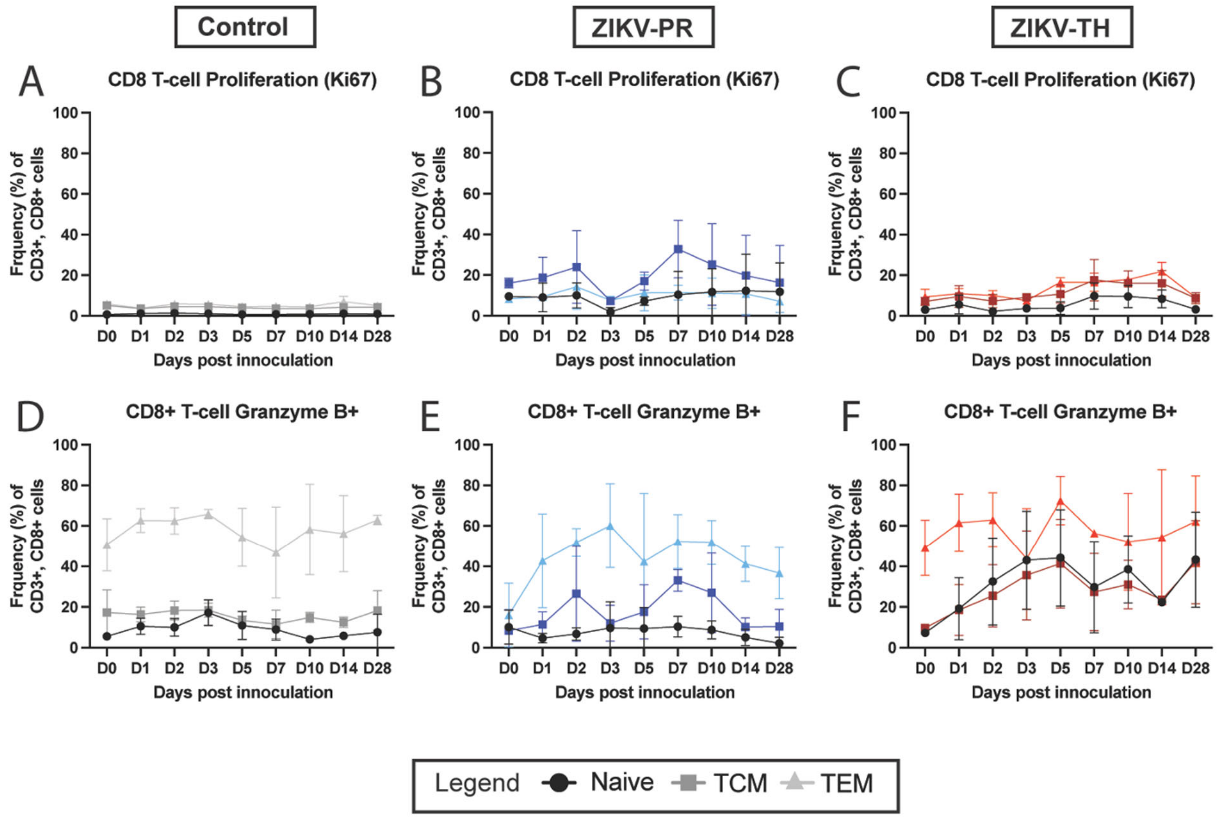

Figure S6 Peripheral Blood CD8 T cell proliferation and granzyme B expression following ZIKV inoculation. Rhesus macaque PBMC from indicated time points post-inoculation were analyzed for T cell phenotype using flow cytometry. Proliferating naïve, central memory, and effector memory CD8+ T-cell (A-C) proliferation and (D-F) granzyme B expression (granzyme B+) are shown for control, ZIKV-PR and ZIKV-TH groups. Longitudinal changes in proliferating (Ki67+) and activated (Granzyme B+) cells in the peripheral blood were analyzed using a mixed model approach with a Greenhouse–Geisser correction followed by Tukey's multiple comparisons of control animals to ZIKV infected for each day post-inoculation; for this analysis for this analysis all time points indicated ns $p > 0.05$ (not shown on figure).
